# Comparison of carcass and meat quality traits between lean and fat Pekin ducks

**DOI:** 10.5713/ajas.19.0612

**Published:** 2019-12-24

**Authors:** Si-Ran Ding, Guang-Sheng Li, Si-Rui Chen, Feng Zhu, Jin-Ping Hao, Fang-Xi Yang, Zhuo-Cheng Hou

**Affiliations:** 1 National Engineering Laboratory for Animal Breeding, Key Laboratory of Animal Genetics, Breeding and Reproduction of the Ministry of Agriculture, College of Animal Science and Technology, China Agricultural University, Beijing 100193, China; 2 Beijing Golden Star Duck Center, Beijing 100076, China

**Keywords:** Pekin Duck, Carcass Traits, Meat Quality, Fatty Acids, Intramuscular Fat

## Abstract

**Objective:**

According to market demand, meat duck breeding mainly includes 2 breeding directions: lean Pekin duck (LPD) and fat Pekin duck (FPD). The aim of the present study was to compare carcass and meat quality traits between 2 strains, and to provide basic data for guidelines of processing and meat quality improvement.

**Methods:**

A total of 62 female Pekin ducks (32 LPDs and 30 FPDs) were slaughtered at the age of 42 days. The live body weight and carcass traits were measured and calculated. Physical properties of breast muscle were determined by texture analyzer and muscle fibers were measured by paraffin sections. The content of inosine monophosphate (IMP), intramuscular fat (IMF) and fatty acids composition were measured by high-performance liquid chromatography, Soxhlet extraction method and automated gas chromatography respectively.

**Results:**

The results showed that the bodyweight of LPDs was higher than that of FPDs. FPDs were significantly higher than LPDs in subcutaneous fat thickness, subcutaneous fat weight, subcutaneous fat percentage, abdominal fat percentage and abdominal fat shear force (p<0.01). LPDs were significantly higher than FPDs in breast muscle thickness, breast muscle weight, breast muscle rate and breast muscle shear force (p<0.01). The muscle fiber average area and fiber diameter of LPDs were significantly higher than those of FPDs (p<0.01). The muscle fiber density of LPDs was significantly lower than that of FPDs (p<0.01). The IMF of LPDs in the breast muscle was significantly higher than that in the FPDs (p<0.01). There was no significant difference between the 2 strains in IMP content (p>0.05). The polyunsaturated fatty acid content of LPDs was significantly higher than that of FPDs (p<0.01), and FPDs had higher saturated fatty acid and monounsaturated fatty acid levels (p<0.05).

**Conclusion:**

Long-term breeding work resulted in vast differences between the two strains Pekin ducks. This study provides a reference for differences between LPD and FPD that manifest as a result of long-term selection.

## INTRODUCTION

Pekin duck, a world-famous meat duck breed, shows advantageous characteristics such as fast growth, high feed conversion rate, high reproductive rate and strong disease resistance [1]. Pekin ducks account for about 70% of the annual meat duck production in Asia. In China, there are kinds of dishes that use duck as the raw material, each of which requires different traits of duck. Specifically, roasting requires more fat deposition, braising requires higher meat quality, and soup requires more flavor. To satisfy the different preferences of consumers, the original Pekin duck was selected in 2 different directions: lean Pekin duck (LPD) and fat Pekin duck (FPD) [2,3]. For more than 30 years in China, selection of LPD mainly focused on various carcass traits, including breast muscle yield, feed conversion ratio, and growth rate, while in FPD the selection focused on fat traits, including subcutaneous fat ratio and skin thickness. LPD mainly provides meat and its byproducts after slaughter, which has a huge market prospect. FPD is used mainly as raw material for roast duck because of its high subcutaneous fat percentage. In China and Southeast Asia, roast Pekin duck is a classic example of the utilization of the Pekin duck’s excellent subcutaneous fat trait utilization [1]. However, the intensive selection on growth rate has resulted in a decline in meat quality and poor flavor characteristics in meat animals [4]. Improving meat quality became a high priority in the breeding of meat duck.

Meat quality is a comprehensive set of characteristics that is composed of multiple traits, including appearance quality traits, eating quality traits and reliance quality traits [5]. Eat quality traits are series of traits affecting the taste and flavor of meat, such as muscle shear force, water loss rate, intramuscular fat (IMF), and inosine monophosphate (IMP). IMF content of the muscle affects the texture and taste of the meat. An increase in IMF content will improve the juiciness, tenderness, water-holding capacity and flavor of the meat [6,7] and is one of the main flavor-determining compounds in poultry muscle. IMP is a substance produced by the decomposition of ATP in the muscles after the death of the animal, which makes the muscle tasty. In addition, sodium glutamate in the muscle can have a make a synergistic effect with IMP, which makes the fresh flavor stronger [8,9]. Duck meat also contains a large number of polyunsaturated fatty acids (PUFAs) [10], while the high essential fatty acids content of duck meat makes it suitable for human health purposes [11]. The muscle fiber is an important component of breast muscle that determines meat quality. The type and diameter of muscle fiber are related to muscle tenderness [12]. In spite of the importance of the issue there are very few studies available on the quality of duck meat.

The objective of this study is to assess the main differences in the carcass and meat quality of 2 different types of Pekin ducks on the market and provide guidelines for breeding, production, and processing of duck meat.

## MATERIALS AND METHODS

### Ducks and rearing system

A total of 62 female Pekin ducks (32 LPDs and 30 FPDs) were reared in Beijing Golden Star Duck Company. *Ad libitum* feeding and drinking were provided from hatching until the end of the study. The animals were fed a starter diet (from 1 day to 3 weeks of age) containing 19% crude protein, 12.81 MJ/kg dietary metabolizable energy (ME), and a grower diet (from 4 weeks of age till the end of the experiment) containing 17.1% crude protein and 11.95 MJ/kg ME. The management and feeding conditions for the LPD and FPD were the same. The feed content and lighting program was the same as in our previous studies [13,14]. All the duck experiments were reviewed and approved by the Animal Care and Use Committee of China Agricultural University with approval number SYXK 2007-0023.

### Slaughter and carcass performance

The experimental ducks were slaughtered at 42 days of age, following fasting for 12 h prior to slaughter, during which time drinking water was provided freely. Slaughter was carried out according to the standard meat duck slaughtering process. Live body weight, carcass weight, eviscerated weight, breast muscle weight, leg muscle weight, abdominal fat weight and subcutaneous fat weight of the animals were measured. The thickness of subcutaneous fat and breast muscle was measured using an ultrasonic method [14]. The heart, liver, gizzard, abdominal fat, subcutaneous fat and breast muscle were weighed for each duck. The eviscerated weight was obtained by removing the weight of viscera (heart, liver, spleen, gizzard, intestines, crop, esophagus, trachea and reproductive organs), head and feet. The slaughter traits were calculated according to the method described previously [14,15].

### Physical properties of breast and subcutaneous fat

Shear force was used as a representative of meat tenderness. The left breast muscle and subcutaneous fat were used to determine the shear force and free water loss rate. Shear force and free water loss rate were measured by using a texture analyzer (model XTplus Stable Micro Systems, Surrey, UK). The muscle was cut parallel to the longitudinal orientation of muscle fibers and shear force was recorded by means of the texture analyzer (test speed 2 mm/s; displacement 98% thickness; trigger force 5 g). The water holding capacity (WHC) was measured as weight loss during constant pressure. Using a pair of blades perpendicular to the direction of the muscle fiber to cut the breast muscle with a 1.0 cm thickness, a sharp circular sampler with a diameter of 2.5 cm (circular area is 5 cm) was used to cut out a sample from the center part and weigh m1. After weighing, the muscle sample was wrapped in gauze and placed in filter paper, placed on the texture analyzer, pressurized to 35 kg for 5 min. Immediately after removal of the pressure, it was weighed (m_2_) and the water loss rate was determined as X = (m_1_−m_2_)/m_1_×100%.

**Figure 1:**
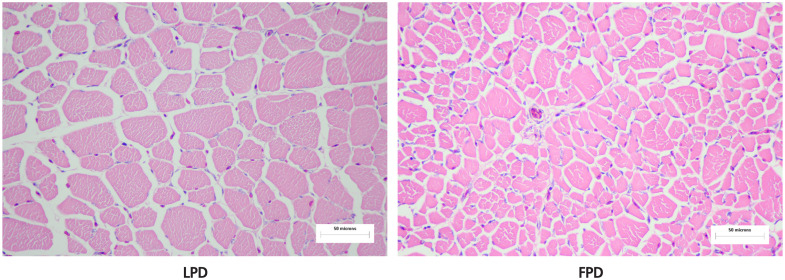
Paraffin section of breast muscle of lean Pekin duck (LPD) and fat Pekin duck (FPD) (400× magnification). This is a cross-sectional view of the pectoral muscle of 2 strains of Pekin duck. The red parts of the picture are a cross section of the breast muscle fibers.

For measuring muscle fiber diameter, breast meat samples (volume: 2 cm×1 cm×1 cm) were cut along the same part of muscle fibers and stored in formalin solution for 48 h. After being washed with tap water, they were dehydrated in an ethanol series. The meat samples were immersed in xylene: ethanol (1:1) solution and then embedded in paraffin. Paraffin sections (10 μm) were cut and stained with hematoxylin and eosin. The sections were photographed with a microscope at 400× magnification (Figure 1) and histologically analyzed with Image-Pro Plus version 6.0 software to determine the average area of muscle fibers, average diameter, average density and the ratio of length diameter to width diameter.

### Inosine monophosphate in the breast muscle

Samples were trimmed of all visible connective tissue and fat. The right breast muscle was made into a meat paste and stored at −20°C. Meat samples (approximately 5 g) were homogenized with 20 mL 5% perchloric acid and centrifuged at 4,000 rpm at 10°C. The supernatant was diluted with 15 mL 5% perchloric acid and filtered. The pH of the filtrate was adjusted to 6.5 with sodium hydroxide solution, adjusted to 100 mL, filtered using a 0.22 μm membrane and the IMP content was determined by high-performance liquid chromatography.

### Intramuscular fat in the breast muscle

Hydrochloric acid solution (2 mol/L) was added to 3 g of breast muscle paste prepared previously and the mix was gently boiled for 1 h. It was then washed after drying at 103°C for 1 h, using the Soxhlet extraction method [16]. The IMF content was calculated by dividing the weight of fat by the total weight of the sample.

### Fatty acids in the breast muscle

Before the determination of fatty acid composition, duck breast muscle stored at −20°C was taken out of the freezer before measuring fatty acids and then frozen under vacuum at −20°C for 72 h. For the determination of fatty acid composition, the sample was added into a 4 mL reaction bottle. The fatty acid was methylated by 6 mL of 10% acetyl chloride methanol (acetyl chloride has a volume of 10% of methanol) solution, then the sample was filled with nitrogen, and a water bath was conducted at 80°C for 2 h. After 1 mL normal hexane was added into the reaction system and shaken, 1.5 mL of 6% potassium carbonate solution was added. After shaking, the supernatant was taken into the sample bottle and sent to the automatic gas chromatography for determination. The analysis was performed on an Agilent 7890 gas chromatograph with a flame ionization detector, using a DB-23 column (60 m ×250 μm×0.2 μm) for detecting fatty acid methyl esters (FAME). The initial temperature of the oven was set at 50°C for 2 min, rising to 175°C for 3 min at a speed of 25°C per minute. Then the column temperature was raised at 5°C/min to 200°C, and immediately to 210°C at a rate of 1.5°C/min. Finally, it rises to 230°C at 2°C/min where it remained for 6 min. The detector and injector were both at 250°C and the flow rate was set at 1.2 mL/min. The peak of FAMEs was identified by comparison with the similar retention time and quantified for the 37 component FAMEs mix (Supelco, 37 Component FAME mix C4–C24) as an external marker (analyzed by the GC Chem Station software of Agilent, Palo Alto, CA, USA).

### Statistical analysis

Statistical analyses were performed using the R statistical software (version 3.4.3). Differences between the 2 strains of Pekin ducks were tested by Student’s *t*-test. Spearman’s simple correlation between the different traits was calculated. The principal component analysis (PCA) was used to analyze the dimension cluster of the measured 12 carcass and meat quality traits. The purpose of using PCA was to condense the original information into a smaller number of representative traits to discriminate the 2 lines and also explore relationships among traits. Differences were considered significant at the p<0.05 or p<0.01 level.

## RESULTS

### Comparison of slaughter performance between LPD and FPD

The live body, carcass, and eviscerated weight of the LPD were significantly higher than those of the FPD (p<0.01) at the age of 42 days (Table 1). LPD had a significantly higher eviscerated rate than FPD (p<0.05). The carcass rate was similar between the 2 lines (p>0.05).

### Comparison of fat properties between LPD and FPD

Fat-related traits of the 2 strains are shown in Table 2. The results indicate that the thickness of the subcutaneous fat, subcutaneous fat weight, subcutaneous fat percentage, abdominal fat percentage and subcutaneous fat shear force of the FPD were significantly higher than those of the LPD (1.76 mm, 668.73 g, 35.81%, 2.62% and 12.18 N vs 1.23 mm, 562.34 g, 23.64%, 1.21% and 7.91 N, respectively, p<0.01). There was no significant difference in the density of pores between the 2 strains (p>0.05).

### Comparison of physical properties between LPD and FPD breast muscle

The physical properties of the breast muscles are shown in Table 3. The thickness of breast muscle, breast muscle weight, breast muscle rate and the shear force of breast muscle were significantly higher in 42-day-old LPDs than FPDs (19.67 mm, 455.72 g, 19.14%, 15.78 N vs 10.43 mm, 182.73 g, 9.81%, 10.75 N, respectively, p<0.01). There was no significant difference in the water loss rate between the 2 groups (p>0.05). The average area of muscle fiber and the diameter of muscle fiber in the LPD group were significantly higher than those in the FPDs (378.42 vs 149.34 μm^2^ and 28.02 vs 17.76 μm, respectively, p<0.01). The density of muscle fiber was significantly in the LPD than in the FPD group (p<0.01), but there was no significant difference in the ratio of the longer diameter to the shorter diameter of muscle fiber (p>0.05). Finally, based on the paraffin sections, the muscle fibers of FPDs were clearly denser, and the diameter and area of muscle fibers were also significantly smaller than those of LPDs.

### Comparison of the content of IMP, IMF and fatty acids in breast muscle between LPD and FPD

The contents of IMP and IMF are shown in Table 4, and the composition of fatty acids are presented in Table 5. No obvious difference in the IMP content of breast muscle was found between LPDs and FPDs (0.85 vs 0.79 mg/g, p>0.05), while a significant difference was found in the IMF content of breast muscle between the 2 groups (1.44% vs 1.22%, p<0.01). The composition of fatty acids in the breast muscle in the 2 strains were similar, with the top five fatty acids being arachidonic acid (C20:4, 23% to 24%), oleic acid (C18:1, 19% to 20%), linoleic acid (C18:2, 14% to 16%), palmitic acid (C16:0, 12%) and stearic acid (C18:0, 11%), accounting for more than 80% of the total fatty acids. The PUFA content of the LPD was significantly higher than that of the FPD (p<0.01).

### Correlation analysis between different traits

The result of correlation analysis is shown in Table 6. IMF content of the breast muscle is positively correlated with live body weight and the breast muscle rate (r = 0.39 and r = 0.32, respectively, p<0.01), and is negatively correlated with the subcutaneous fat percentage and the abdominal fat percentage (r = −0.36 and r = −0.33, respectively, p<0.01). The diameter of muscle fiber showed a positive correlation with the IMP content (r = 0.27, p<0.05). In addition, it is also negatively correlated with the IMF content (r = −0.32, p<0.01).

### Principal component analysis

**Figure 2:**
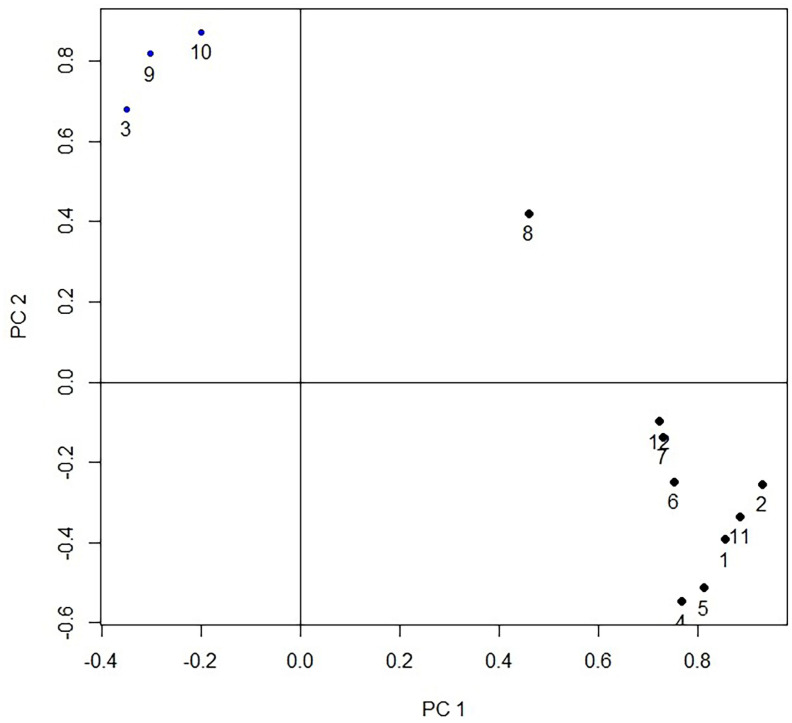
Projection of the carcass traits measurements by the 2 first principal components. 1 = live body weight; 2 = carcass weight; 3 = thickness of subcutaneous fat; 4 = thickness of breast muscle; 5 = breast muscle weight; 6 = heart weight; 7 = liver weight; 8 = gizzard weight; 9 = abdominal fat weight; 10 = subcutaneous fat weight; 11 = eviscerated weight; 12 = head weight.

**Figure 3:**
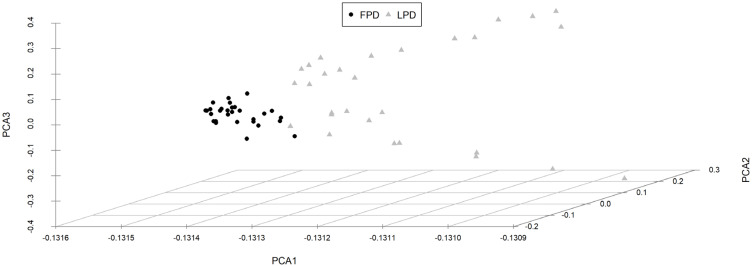
Projection of 1 individual of each strain in the plane defined by the three principal components. Comp.1, Comp.2, Comp.3 represent the first, second and third principal component, respectively. Each point in the figure represents 1 individual.

The first three principal components (PC1, PC2, and PC3) with eigenvalues higher than 1 which were extracted from the 12 original traits, can explain the 81.27% cumulative variance. While the PC1 predominates account for 60.17% of the cumulative variance. There were three groups of variables in the projection of first two principal components (Figure 2). The first group includes the fat-related traits (thickness of subcutaneous fat, abdominal fat weight, subcutaneous fat weight); the second group includes the gizzard while the third group includes the remaining 8 traits (live body weight, carcass weight, thickness of breast muscle, breast muscle weight, heart weight, liver weight, head weight). The results indicate that the current 12 carcass traits could be divided into three categories. In PC1, the vector projection of the thickness of subcutaneous fat, abdominal fat weight, and subcutaneous fat weight, indicates that fat traits are the most important traits that can discriminate the different strains (Figure 2). The projection of the individual of 2 strains in the plane defined by the three principal components is shown in Figure 3, which indicates that FPDs and LPDs are clustered together, and the distribution area of the 2 strains has a clear line of demarcation.

## DISCUSSION

The yield and meat quality are important factors influencing the sale price and consumer preference. Compared with broilers, Pekin duck has a significantly higher eviscerated rate and has more meat value [17,18]. In the present study, the slaughter rate of Pekin ducks was higher than 80%, and the eviscerated rate was higher than 65%. This showed that both LPD and FPD had excellent meat production performance. The high subcutaneous fat weight, subcutaneous fat percentage, and subcutaneous fat thickness are the most important traits for the FPD line, which is normally preferred as raw material for roast Pekin duck. There was a significant difference between the 2 strains in the shear force of subcutaneous fat, which is due to selective breeding for the subcutaneous fat thickness of the FPD. Additional work is needed to improve the IMF content of duck breast muscle. After a long period of divergent selection, FPD and LPD lines show significant differences in carcass traits, especially fat-related traits. Interestingly, the gizzard weight was used as the third independent trait while we found no difference between the 2 strains. This suggests that artificial selection on growth and fat deposition does not have a significant large effect on gizzard weight.

IMP, as a substance that contributes to the determination of meat flavor, plays an important role in giving the umami taste of muscle and is also one of the important flavor indexes [8]. IMP has a great influence on the taste of breast meat. Different studies found great variations in IMP content, both within and among species. IMP content from various studies in chickens ranged from 1.40 to 1.81 mg/g [19,20]. A few studies available in ducks also showed large variations in IMP content. Cherry Valley ducks had about 1.55 mg/g IMP in the breast muscle [21]. However, since IMP content is greatly influenced by nutrition, breeds, and storage time, more work should be performed from both of those selection aspects. The limited number of studies about the heritability of IMP in chicken and other species showed that IMP has low heritability [22]. In the experimental population, the IMP content showed a large coefficient of variance (12.7% and 17.6%), indicating that IMP had a possibility for improvement. Studies have shown that tenderness and flavor of duck breast are related to the IMF content [23]. However, we found no significant correlation between tenderness and IMF content in this study. IMF content in the breast muscle did not correlate with subcutaneous fat weight either. In addition, we found a significant negative correlation between IMF and other fat traits (subcutaneous fat and abdominal fat), which may indicate that the deposition of IMF is different from that of subcutaneous fat. These results may have an important impact on duck breeding when considering IMF in the breast muscle and body fat content.

The composition of fatty acids is also an important factor affecting the meat flavor. Excessive intake of saturated fatty acid (SFA) is believed to lead to a series of diseases in humans, and the higher the unsaturated fatty acid content in the meat, especially PUFAs, the more beneficial effect on human health can be expected. A previous study suggested that the PUFA: SFA (P:S) ratio in food intake was determined to be above 0.4 [24]. The P:S ratio in Pekin duck was close to 2 in this study, which is considered suitable for human health purposes. However, the n-3 PUFA content in our study was less than that reported by others [25,26]. Although there are some differences between the 2 strains, the composition of total fatty acids was similar in general between two strains. Compared to the content and composition of red meat (pork, beef, and mutton) found in other studies, Pekin duck showed clear differences compared to those meat types in terms of fatty acids composition. In general, red meat has a high proportion of SFA, resulting in a low ratio of P:S (0.3 to 0.4); C18:1 and C16:0 are dominant in red meat [24]. By contrast, the fatty acids in Pekin duck breast muscle are mainly unsaturated fatty acids and C20:4 is the major component of total fatty acids [27–31].

LPD has much higher breast muscle yield than FPD. The selection has also led to large differences in various muscle quality-related traits between the two lines. Tenderness is thought to be an important index for discriminating the meat quality. It was reported that the diameter of muscle fibers affects the tenderness of the muscle [5]. A previous study showed that there was a significant positive correlation between the diameter of breast muscle fibers and the shear force in broilers [32]. The results of the present study support those findings. FPD has more tender breast muscles, which is regarded as more suitable for the traditional roast duck. It was shown previously that increasing the area of muscle fibers make the meat darker and causes a higher WHC in broilers [33], while muscle growth mainly increases the size of muscle cells [3]. In the present research, the interval between the breast muscle fibers as well as the size of the muscle fibers was larger in LPD compared to those in FPD, and it is speculated that there may be more connective tissue and adipocytes between the muscle fibers in the LPD strain. The IMF in the LPD breast muscle was also higher than in the FPD, which also supports the hypothesis that there may be more adipocytes between the muscle fibers in the LPD. However, more work is needed to test this hypothesis.

In conclusion, the long-term selection of fat-related traits and body growth-related traits resulted in distinct differences in meat characteristics between the 2 duck lines. There were significant differences in growth rate, subcutaneous fat percentage, shear force, and other indicators. The P:S ratio in both types of duck meat is high, which makes it an ideal meat-type from a dietary standpoint. This study provided general carcass and meat quality parameters for the 2 most popular Pekin duck strains.

## Figures and Tables

**Table 1:** Comparison of slaughter performance between LPD and FPD

Traits	LPD	FPD
Live body weight (g)	3,402.19±206.51^A^	2,781.47±136.75^B^
Carcass weight (g)	3,000.27±185.07^A^	2,461.23±123.94^B^
Eviscerated weight (g)	2,378.25±146.77^A^	1,863.63±103.61^B^
Carcass rate (%)	82.89±21.76	88.73±6.76
Eviscerated rate (%)	69.93±2.03^a^	67.20±5.45^b^

LPD, lean Pekin duck; FPD, fat Pekin duck.

A,B Different upper case letters as superscripts in the same row indicate very significant differences (p<0.01).

a,b Different lower case letters denote significant differences (p<0.05).

**Table 2:** Comparison of fat properties between LPD and FPD

Traits	LPD	FPD
Thickness of subcutaneous fat (mm)	1.23±0.37^B^	1.76±0.31^A^
Subcutaneous fat weight (g)	562.34±61.12^B^	668.73±72.02^A^
Subcutaneous fat percentage (%)	23.64±2.06^B^	35.81±2.22^A^
Abdominal fat percentage (%)	1.21±0.50^B^	2.62±0.43^A^
Subcutaneous fat shear force (N)	7.91±2.55^B^	12.18±2.42^A^
Density of pores (N/cm^2^)	9.72±1.19	10.03±0.92

LPD, lean Pekin duck; FPD, fat Pekin duck.

A,B Different superscripts in the same row indicate significant differences (p<0.01).

**Table 3:** Comparison of physical properties between LPD and FPD breast muscle

Traits	LPD	FPD
Breast muscle thickness (mm)	19.67±2.03^A^	10.43±1.03^B^
Breast muscle weight (g)	455.72±49.47^A^	182.73±18.26^B^
Breast muscle rate (%)	19.14±1.38^A^	9.81±0.88^B^
Breast muscle water loss (%)	39.52±2.05	40.54±2.01
Breast muscle shear force (N)	15.78±4.12^A^	10.75±1.48^B^
Area of muscle fiber (μm^2^)	378.42±98.37^A^	149.34±28.57^B^
Diameter of muscle fiber (μm)	28.02±3.71^A^	17.76±1.68^B^
ratio of the longer to the shorter diameter	1.78±0.07	1.81±0.06
Density of muscle fiber (N/mm^2^)	1,368.74±349.16^B^	2,856.58±429.88^A^

LPD, lean Pekin duck; FPD, fat Pekin duck.

A,B Different superscripts in the same row indicate significant differences (p<0.01).

**Table 4:** Comparison of inosine monophosphate and intramuscular fat content of breast muscle between LPD and FPD

Traits	LPD	FPD
IMP (mg/g)	0.85±0.15	0.79±0.10
IMF (%)	1.44±0.40^A^	1.22±0.20^B^

LPD, lean Pekin duck; FPD, fat Pekin duck; IMP, inosine monophosphate; IMF, intramuscular fat.

A,B Different superscripts in the same row indicate significant differences (p<0.01).

**Table 5:** Comparison of fatty acids in breast muscle

Fatty acids	Strain		Fatty acid	Strain	
	
	LPD	FPD		LPD	FPD
C4:0	0.01±0.003	0.01±0.002	C16:1	0.18±0.08	0.18±0.07
C6:0	0.01±0.004	0.01±0.004	C17:1	0.16±0.03^B^	0.19±0.02^A^
C11:0	0.02±0.004	0.02±0.004	C18:1	5.70±2.05	5.82±1.70
C14:0	0.06±0.03	0.05±0.02	C20:1	0.14±0.03^A^	0.13±0.020^B^
C15:0	0.01±0.01^a^	0.01±0.003^b^	C24:1	0.13±0.02^B^	0.23±0.05^A^
C16:0	3.47±0.75	3.46±0.51	MUFA	7.16±2.14	7.56±1.80
C17:0	0.14±0.03^A^	0.11±0.01^B^	C18:2	4.78±1.13^A^	4.11±0.60^B^
C18:0	3.36±0.36	3.27±0.19	C18:3	0.08±0.04^a^	0.06±0.02^b^
C20:0	0.09±0.01^B^	0.10±0.01^A^	C20:2	0.81±0.12	0.85±0.11
C21:0	0.49±0.14	0.55±0.12	C20:3	0.81±0.11^b^	0.87±0.10^a^
C22:0	0.04±0.01^A^	0.03±0.01^B^	C20:4	6.89±0.61^A^	6.31±0.52^B^
C23:0	0.02±0.01	0.02±0.01	C20:5	0.07±0.01^B^	0.08±0.01^A^
C24:0	0.17±0.01^A^	0.16±0.01^B^	C22:2	0.02±0.01^B^	0.03±0.01^A^
SFA	7.89±1.20	7.79±0.75	C22:6	0.05±0.01^B^	0.06±0.01^A^
C15:1	0.86±0.08^B^	1.01±0.07^A^	PUFA	13.50±1.46^A^	12.36±0.79^B^

The values are dry matter content.

LPD, lean Pekin duck; FPD, fat Pekin duck; MUFA, monounsaturated fatty acid; SFA, saturated fatty acid; PUFA, polyunsaturated fatty acids.

A,B Different upper case letters as superscripts in the same row indicate strong significant differences (p<0.01).

a,b Different lower case letters denote significant differences (p<0.05).

**Table 6:** Correlation of some traits in Pekin ducks

Items	IMF	Area of muscle fiber	Diameter of muscle fiber	Ratio of the longer to the shorter diameter	Density of muscle fiber
Live body weight	0.39^**^	0.79^**^	0.81^**^	−0.15	−0.85^**^
Carcass rate	−0.16	−0.11	−0.13	−0.23	0.18
Eviscerated rate	−0.1	0.26^*^	0.25^*^	−0.28^*^	−0.19
Subcutaneous fat percentage	−0.36^**^	−0.8^**^	−0.84^**^	0.11	0.86^**^
Breast muscle rate	0.32^**^	0.87^**^	0.9^**^	−0.11	−0.91^**^
Abdominal fat percentage	−0.33^**^	−0.77^**^	−0.8^**^	0.07	0.8^**^
Breast water loss	−0.04	−0.21	−0.22	−0.04	0.17
Breast shear force	0.12	0.6^**^	0.59^**^	−0.26^*^	−0.58^**^
IMP	0.24	0.24	0.27^*^	0.04	−0.25^*^
IMF	1	0.27^*^	0.32^**^	0	−0.33^**^

IMP, inosine monophosphate; IMF, intramuscular fat.

** Denotes significant level at p<0.01, and

* indicates significant level at p<0.05.
